# Sanatoria: Who Is to Pay?

**Published:** 1901-09-28

**Authors:** 


###  The Hospital

A Journal of

The Medical Sciences and Hospital Administration.

Vol. XXX.—No. 783.] Edited by Sir Henry Burdett, K.C.B., and Solomon C. Smith, M.D., M.R.C.P. [September 28, 1901.

### SANATORIA: WHO IS TO PAY?

Knowing what we now know in regard to the advantages which may be derived from the treatment of certain cases of phthisis in sanatoria, the very serious question arises: Who is to pay? The wealthy, of course, must look after themselves. Going to the other extreme the more we study the distribution of phthisis the more convinced we become that it is a disease especially connected with poverty. Moreover it is in the homes of the poor, far more than among those who are better off, that consumption spreads by infection; hence, whether we look upon the sanatorium treatment of consumption as a means of cure or of prevention, it must always be the case that for many the treatment must be free. For the wealthy and for the poor then all is plain sailing. For the wealthy the laws of supply and demand will at once do all, and perhaps more than all that is necessary; while for the poor the rates will almost of a certainty be called upon to provide the needful. For the very poor indeed the guardians are already responsible, but we do not think that the provision of sanatoria, even for them, should be allowed to become a poor law affair. What we want above all things is to get the poor consumptive out of his home, and to prevent him living out the remainder of his life in cottage surroundings, coughing and spitting and sowing the seeds of the disease among those who are working for his maintenance; and this cannot be done if the rate-supported sanatorium is to be a poor law institution. It is a matter for county councils and sanitary authorities rather than for unions. Between these two extremes, then—*i.e.*, between the rich and the destitute —there lies the field for charity, in assisting those who can pay a little but who cannot pay all. Many of these people can pay something, they are socially quite above those who may properly come upon the rates, and yet they are often quite unable, even with the assistance of their friends, to pay the charges of the fully self-supporting sanatoria.

As Sir Chrichton Browne said, when addressing the International Congress on Tuberculosis, "It is for governesses, curates, clerks, young barristers, and medical men and civil servants, and for the wives and daughters of professional men, of farmers and small tradesmen that sanatoria should be provided by the subscriptions of the charitable or by the assistance of well-disposed capitalists who are willing to do a good work and receive a very small return for their money." Many of these people could pay something, while most of them, if told to go either to the rate-supported institutions or to the five-guineas a-week sanatorium, would certainly remain at home and drift into confirmed consumption.

It seems to us that it is very important that from the very first the proper line should be taken in regard to this question of payment, so that we may avoid in regard to consumption sanatoria the charges of "abuse" which are so often and so thoughtlessly levelled at our great hospitals. To a very large extent such abuse as does exist at the great hospitals arises from the fact that their constitution as charities makes it difficult for them to accept direct and open payment from patients. Under such circumstances it is not to be wondered at that patients, thinking that their lives depend on their getting into hospital, determine to get in at all hazards—even at the cost of pride and perhaps of honesty. Much more will this be the case in regard to sanatoria if they are opened on the same system, and if in deference to the old cry that they are "pure charities" they decline to accept payment from their patients.

Our very conception of the word "charity" requires modification. The mediæval idea that it has something to do with giving pennies to beggars in the street still clings to us, as also does the notion that it is an "abuse of charity" for those who are not destitute to receive its aid. As a fact those who are really destitute ought by rights to be taken charge of by the guardians and charity should bestow itself upon those for whom there is otherwise no provision.

This is especially so in regard to consumption sanatoria. The charity of England is said to be unbounded, and perhaps it is. But we doubt its being large enough, or steady enough, to undertake the charge of the whole mass of the consumptives in the country. The task is too big. Let us then take care from the beginning that its field is properly limited; that those who are now, or would very shortly be upon the rates, be taken altogether by the rates; that the rich be left to themselves; and that those between the extremes become the charge of the charitable. In so dividing the work do not let us run away with the notion that the receiving of charity is incompatible with self-help and self-respect. It is no doubt a charitable action to take a consumptive pauper by the hand and as a free gift to coddle him up so that he shall at least live on and bear his miserable burden for another year. But it is a still higher charity to help a struggling worker to a means of cure which without such help he could not obtain; and to do this is still a charity even though the recipient should pay perhaps the larger portion of the cost of his treatment. With the work so divided we think the charity of England ought to suffice; but if it is to do so it must draw the line somewhere, and must take care not to squander its resources either in saving the rates or in the establishment of purely eleemosynary institutions for the use of those who could well afford to contribute something towards their own support.

